# Pioglitazone inhibits the proliferation and metastasis of human pancreatic cancer cells

**DOI:** 10.3892/ol.2014.2553

**Published:** 2014-09-23

**Authors:** ITASU NINOMIYA, KEISUKE YAMAZAKI, KATSUNOBU OYAMA, HIRONORI HAYASHI, HIDEHIRO TAJIMA, HIROHISA KITAGAWA, SACHIO FUSHIDA, TAKASHI FUJIMURA, TETSUO OHTA

**Affiliations:** Gastroenterologic Surgery, Department of Oncology, Division of Cancer Medicine, Graduate School of Medical Science, Kanazawa University, Kanazawa, Ishikawa, Japan

**Keywords:** pioglitazone, lymph node metastasis, lung metastasis, pancreatic cancer, differentiation, rectal xenograft model

## Abstract

Proliferator-activated receptor-γ (PPAR-γ) is a nuclear receptor that acts as a transcription factor in several types of tissue. PPAR-γ ligands are known to inhibit numerous cancer cell processes, including pancreatic cancer cell proliferation through terminal differentiation. Previous studies concerning the inhibitory effect of PPAR-γ ligands derived from thiazolidinediones (TZDs) on the metastatic potential of cancer cells have been reported. The present study aimed to investigate whether pioglitazone, a prescription TZD class drug and a ligand of PPAR-γ, inhibits the proliferation and metastasis of pancreatic cancer cells. The inhibitory effect of pioglitazone on the proliferation of the Capan-1, Aspc-1, BxPC-3, PANC-1 and MIApaCa-2 pancreatic cancer cell lines was analyzed. Alterations in carcinoembryonic antigen (CEA), interleukin-8 (IL-8) and cyclooxygenase-2 (COX-2) mRNA expression levels subsequent to pioglitazone treatment were examined in BxPC-3 cells by quantitative reverse transcription polymerase chain reaction. In addition, whether the oral administration of pioglitazone prevents tumorigenesis and spontaneous BxPC-3 cell lymph node and lung metastases was investigated using a rectal xenograft model. Pioglitazone treatment resulted in the inhibition of proliferation in all five pancreatic cancer cell lines *in vitro*. Pioglitazone induced CEA mRNA expression, suppressed IL-8 and COX-2 mRNA expression *in vitro*, and inhibited BxPC-3 xenograft growth. Pioglitazone also reduced BxPC-3 cell lymph node and lung metastasis in the rectal xenograft model. These results suggest that pioglitazone treatment inhibited the proliferation and metastasis of pancreatic cancer cells through the induction of differentiation and the inhibition of angiogenesis-associated protein expression.

## Introduction

Pancreatic ductal adenocarcinoma behaves aggressively and is an important cause of cancer-related mortality. The five-year survival rate following curative surgery has been reported to be 10–20% (1,2). Malignant pancreatic cancer cells are characterized by uncontrolled proliferation, an inability to express the differentiated features of normal duct cells and the rapid invasion of adjacent tissues (3).

Peroxisome proliferator-activated receptor-γ (PPAR-γ) is a ligand-activated transcription factor that belongs to the nuclear hormone receptor super family. Patients who receive PPAR-γ-activating drugs (used to treat several million patients with type 2 diabetes mellitus) are at significantly lower risk of lung cancer (4). The activation of PPAR-γ in non-small cell lung cancer (NSCLC) has been shown to inhibit the proliferation of NSCLC cells *in vitro* and in xenograft models (5–8). Thiazolidinediones (TZDs), such as pioglitazone and rosiglitazone, are a novel class of antidiabetic drugs that attenuate the insulin resistance associated with obesity, hypertension and impaired glucose tolerance in humans, as well as in several animal models of non-insulin-dependent diabetes (9). TZDs have been found to act as ligands for PPAR-γ, a member of the nuclear receptor superfamily of ligand-dependent transcription factors predominantly expressed in adipose tissue (10,11). TZDs have been shown to inhibit the growth of liposarcoma (12) and breast (13,14), colon (15–17), prostatic (18), gastric (19) and pancreatic (20–22) cancer. The proliferation rates of cultured breast and colon cancer cells are reduced by treatment with TZDs. The treatment also causes changes in cell morphology and gene expression that is indicative of a more differentiated state (16,23,24). Consequently, PPAR-γ has become a potential molecular target in the development of anticancer drugs, and TZDs have been submitted for use in differentiation-mediated therapy in PPAR-γ-expressing tumors. Despite promising results obtained from *in vitro* and *in vivo* studies of tumor growth following TZD treatment, few reported studies (24–26) have examined the effect of a PPAR-γ ligand on the metastatic potential of cancer cells in an animal model and the underlying molecular mechanisms. The inhibitory effect of TZD on colon cancer cell metastasis has been previously demonstrated in an animal model (24) with spontaneous lymph node and hematological metastases following intra-rectal injection of tumor cells in the nude mouse rectum. The model is suitable for assessment of the antimetastatic efficacy of novel agents using quantitative polymerase chain reaction (PCR) to amplify cancer-related human β-globin DNA in the target organ (27). PPAR-γ has been shown to be expressed in human pancreatic cancer, and TZDs have been found to inhibit pancreatic cell proliferation *in vitro* (20,28,29). TZDs inhibit the invasiveness of pancreatic cancer (22) and pioglitazone inhibits pancreatic cancer growth *in vivo* (21). However, the effect of TZDs on the metastatic potential of pancreatic cancer cells remains unknown.

The present study aimed to investigate the inhibitory effect of pioglitazone on the proliferation of pancreatic cancer cell lines. In addition, the study used a rectal xenograft model to examine whether the oral administration of pioglitazone prevents tumorigenesis and the spontaneous lymph node and lung metastases of pancreatic cancer.

## Materials and methods

### Cell lines

Capan-1 (well-differentiated adenocarcinoma), Aspc-1 (moderately-differentiated adenocarcinoma), BxPC-3 (moderately-differentiated adenocarcinoma), PANC-1 (poorly-differentiated adenocarcinoma) and MIApaCa-2 (undifferentiated carcinoma) human pancreatic cancer cell lines were obtained from the American Type Culture Collection (Rockville, MD, USA) and cultured in RPMI 1640 medium (Nissui Pharmaceutical Co., Ltd., Tokyo, Japan) supplemented with 10% heat-inactivated fetal bovine serum (JRH Biosciences, Lenexa, KS, USA), and 100 U/ml penicillin and 100 μg/ml streptomycin, at 37°C in a humidified atmosphere of 95% air/5% CO_2_.

### Chemicals

Pioglitazone, donated by Takeda Pharmaceutical Co., Ltd., (Tokyo, Japan), was dissolved in dimethyl sulfoxide (DMSO) and then diluted to the appropriate concentrations with culture medium. The final concentration of DMSO in the medium was ≤0.1% (v/v).

### Cell proliferation assay

The proliferation inhibition of pancreatic cancer cells treated with pioglitazone was determined by a standard 3-(4, 5-dimethylthiazol-2-yl)-2,5-diphenyltetrazolium bromide assay. Each cell line was treated with pioglitazone at various concentrations (0.01, 0.1, 1, 10 and 100 μM) for 48 h. The percentage inhibition was determined by comparing the cell density of the drug-treated cells with that of the untreated control cells.

### Reverse transcription (RT)-PCR

Changes in carcinoembryonic antigen (CEA), interleukin-8 (IL-8) and cyclooxygenase-2 (COX-2) mRNA expression levels subsequent to pioglitazone treatment in BxPC-3 cells were evaluated by quantitative RT-PCR. CEA is a known differentiation marker in pancreatic cancer (30). The present study also focused on IL-8 and COX-2 as angiogenic molecules. The BxPC-3 cells were cultured in medium containing 10 μM pioglitazone. RNA was extracted from the cells using Isogen systems reagents (Nippon Gene Co., Ltd., Tokyo, Japan). Subsequent to heat denaturation at 68°C for 15 min with 500 pmol oligo(dT) primer, 10 μg RNA was reverse-transcribed to first-strand cDNA at 42°C for 60 min in a reverse-transcription solution. This solution contained 400 units Moloney murine leukemia virus reverse transcriptase (Invitrogen Japan K. K., Tokyo, Japan), 50 mm Tris-HCl (pH 8.3), 75 mm KCl, 3 mm MgCl_2_, 0.01 M DTT, 0.5 mm of each dNTP and 16 units RNasin^®^ (Promega Corporation, Madison, WI, USA) to obtain a final volume of 100 μl. Reverse-transcribed cDNA solution corresponding to 100 ng total RNA was amplified by quantitative PCR using a 5′ nuclease assay and an ABI Prism 7700 Sequence Detector (TaqMan; PE Biosystems Japan, Tokyo, Japan). This reaction was conducted in a 50 μl reaction mixture containing 200 nM forward and reverse primers, 100 nM probe specific for the targeted cDNA and TaqMan Universal Master mix (PE Biosystems Japan), which was comprised of Ampli-Taq Gold DNA polymerase, dNTP, dUTP, AmpErase uracil-N-glycosylase and reaction buffer. The PCR reaction was conducted using the ABI Prism 7700 Sequence Detector with thermo-cycler conditions as follows: 50°C for 2 min, 95°C for 5 min, followed by 45 cycles of 95°C for 15 sec and 60°C for 1 min. The data were examined using Sequence Detection software (PE Biosystems Japan). The primers and probes for amplifying CEA, IL-8 and COX-2 mRNA were purchased from PE Biosystems Japan. Internal standard gene expression levels were examined using TaqMan GAPDH control reagents (PE Biosystems Japan). Target gene expression levels were standardized to the internal standard gene expression levels. The relative expression levels of CEA, IL-8 and COX-2 mRNA in the pioglitazone-treated BxPC-3 cells were calculated compared with the levels in the untreated cells.

### Xenograft animal models

Five-week-old nude mice (BALB/cAnNCrj-nu/nu) were obtained from Charles River Japan, Inc. (Kanagawa, Japan). Following at least one week of observation, the mice were 6–7 weeks old when the experiments were conducted. All mice were housed in the Laboratory for Animal Experiments, Research Institute, Kanazawa University School of Medicine (Kanazawa, Japan), under laminar airflow conditions. Housing was temperature-controlled with a 12-h/12-h light/dark cycle. For inoculation, log-phase BxPC-3 cells were harvested with trypsin EDTA, washed three times with RPMI and resuspended in RPMI at 1×10^7^ cells/ml density. The mice were anesthetized with ether and placed in a supine position. A 7-mm incision was made in the anterior end of the anorectal wall in the anorectal region to prevent colonic obstruction due to rectal tumor progression. The BxPC-3 tumor cells suspended in RPMI (1×10^6^ cells/0.1 ml/mouse) were slowly injected submucosally into the posterior wall with a 27-gauge needle. The mice received solute (DMSO) or pioglitazone (20 mg/kg/day) orally by gavage. Treatment was initiated seven days after tumor cell inoculation and was continued five times per week for five weeks. To evaluate the antitumor and antimetastatic effects of pioglitazone, the mice were sacrificed by anesthesia with pentobarbital sodium six weeks after tumor cell inoculation. The antitumor effect of pioglitazone in the primary site was evaluated by measuring the weight of the peri-anorectal tumor. To evaluate metastases, the lungs and the lymph nodes surrounding the iliac artery and abdominal aorta were excised. DNA from each specimen was extracted by the standard proteinase K digestion-phenol chloroform extraction method, as previously described (31). The human β-globin-related sequence in 1 μg of each extracted DNA was amplified with primers and a probe specific for human the β-globin gene using the ABI Prism 7700 Sequence Detector (TaqMan). The concentrations of the reaction components, with the exception of template DNA and the thermo-cycler conditions, were the same as those for the aforementioned cDNA amplification. The number of metastasized tumor cells in the excised whole organ was calculated from the standard curve of a serial dilution series of BxPC-3 DNA, following quantitative PCR, as previously described (27). The primer and probe sequences for human β-globin gene amplification were as follows: Forward, CACTGACTCTCTCTGCTATTGGTC and reverse, AGGAGTGGACAGATCCCCAAA; TaqMan probe, 6FAM5′-CTACCCTTGGACCCAGAGGTTCTTTGAGTC-3′TAMRA. The study design was approved by the local ethics committee for animal experiments at the Takara-machi Campus of Kanazawa University.

### Statistical analysis

The Mann-Whitney U test was used for statistical analysis of the antitumor and antimetastatic effect of pioglitazone in the xenograft model. Student’s t-test was used for statistical analysis of the effect of pioglitazone on cancer cell proliferation and the alteration of mRNA expression in vitro. P<0.05 was considered to indicate a statistically significant difference.

## Results

### Proliferation inhibition effect of pioglitazone in vitro

**Figure 1:**
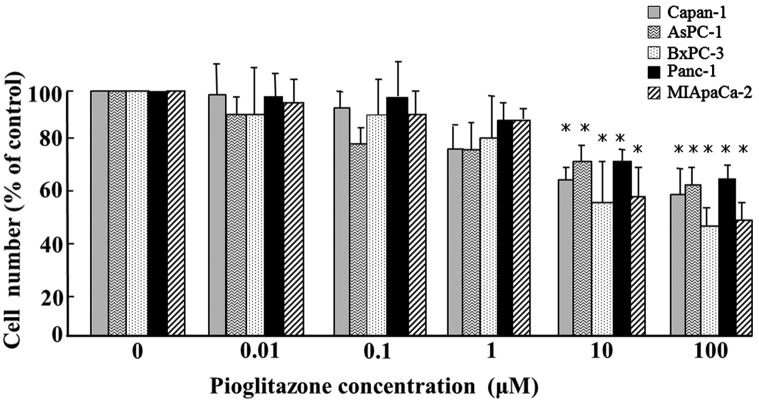
Effect of pioglitazone on pancreatic cancer cell proliferation. The percentage inhibition was determined by comparing the cell density of drug-treated cells to that of untreated controls. Statistical analysis was performed using Student’s t-test. ^*^P<0.05 vs. untreated control.

Pioglitazone significantly inhibited the proliferation of all five pancreatic cancer cell lines (Capan-1, Aspc-1, BxPC-3, PANC-1 and MIApaCa-2) *in vitro* at concentrations >10 μM (P<0.05; Fig. 1).

### Changes in molecular markers following pioglitazone treatment

**Figure 2:**
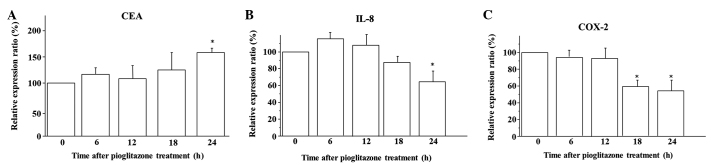
Kinetics of mRNA expression subsequent to pioglitazone treatment. The relative expression level ratio of (A) carcinoembryonic antigen (CEA), (B) interleukin-8 (IL-8) and (C) cyclooxygenase-2 (COX-2) mRNA in pioglitazone-treated BxPC-3 cells was calculated with respect to untreated cells. Statistical analysis was conducted using Student’s t-test. ^*^P<0.05 vs. untreated cells.

The kinetics of mRNA expression subsequent to pioglitazone treatment in the BxPC-3 cells was analyzed by quantitative RT-PCR. Exposure for 24 h to 10 μM pioglitazone induced significant overexpression of CEA mRNA compared with that observed in the untreated cells (P<0.05; Fig. 2A). Furthermore, 10 μM pioglitazone significantly suppressed IL-8 mRNA expression after 24 h of exposure (P<0.05; Fig. 2B) and COX-2 mRNA expression after 18 h of exposure (P<0.05; Fig. 2C).

### Antitumor and antimetastatic effects of pioglitazone in the xenograft model

**Figure 3:**
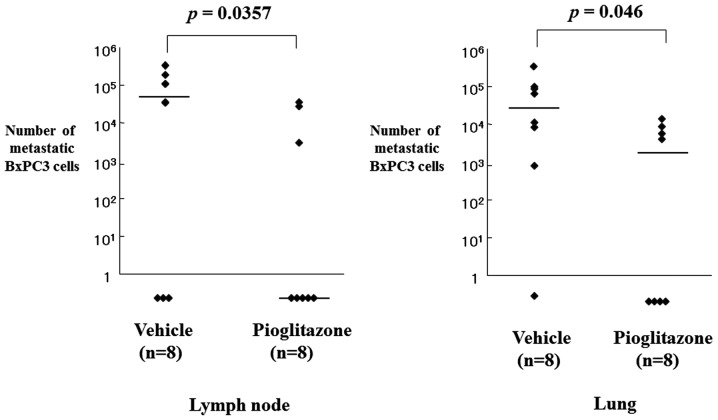
Comparison of metastasized tumor cell numbers in para-aortic lymph nodes and lungs of pioglitazone- and vehicle-treated mice bearing BxPC-3 pancreatic cancer cell rectal xenografts. The number of metastasized tumor cells was calculated by amplification of the human β-globin gene and is presented as the number of tumor cells in the organ. Pioglitazone significantly inhibited lymph node and lung metastases (P=0.0357 and P=0.046, respectively; Mann-Whitney U test).

Macroscopically, the BxpPC-3 xenograft produced a locally aggressive rectal tumor, and subsequently, lymph node metastases surrounding the abdominal aorta appeared six weeks after tumor cell inoculation. Pioglitazone macroscopically inhibited xenograft growth and abdominal lymph node metastasis. The antitumor activity of pioglitazone in the xenograft was examined by comparing the wet weight of each xenograft, and pioglitazone was found to significantly inhibit BxPC-3 xenograft growth by 82.6% (P=0.046; Table I). The antimetastatic activity of pioglitazone was examined by amplifying the human β-globin-related sequence in the lymph nodes and the lungs of rectal xenograft mice. Quantification of cancer metastasis by calculating the number of metastasized tumor cells using the quantitatively-amplified β-globin gene revealed that pioglitazone significantly inhibited lymph node and lung metastasis (P=0.035 and P=0.046, respectively; Fig. 3; Table II).

## Discussion

In the present study, a ligand of PPAR-γ pioglitazone inhibited pancreatic cancer cell proliferation *in vitro* and *in vivo*. Pioglitazone induced pancreatic cancer differentiation with CEA overexpression, and inhibited angiogenic factor IL-8 and COX-2 mRNA expression. Furthermore, pioglitazone prevented spontaneous pancreatic cancer lymph node and lung metastases in a xenograft model.

High PPAR-γ expression levels have been previously identified in human pancreatic cancer cells, and TZD treatment has been shown to inhibit cellular proliferation and induce cellular differentiation (20). In the present study, pioglitazone induced the upregulation of CEA mRNA expression in the BxPC-3 cells. These results indicate that pioglitazone also increased pancreatic cancer cell differentiation, as CEA expression has been previously observed to be associated with the degree of differentiation in pancreatic cancer (30). PPAR-γ activation may also result in the differentiation of pancreatic cancer cells themselves. PPAR-γ ligands inhibit the cellular proliferation of pancreatic cancer, a process comparable with the terminal differentiation induced by cessation of cell proliferation and the accumulation of cells in the G_1_ phase of the cell cycle (20). The detailed mechanism of proliferation inhibition, however, is considered to differ from cell to cell, with the exception of that caused by PPAR-γ.

In the present study, treatment with pioglitazone was demonstrated to inhibit lymph node and lung metastasis, as well as tumor growth in a rectal xenograft model. Several previous studies concerning the metastasis inhibition effect of TZDs have been published (24,25). However, the underlying mechanism of metastasis inhibition by TZDs remains unclear. The data from the present study revealed that pioglitazone suppressed IL-8 and COX mRNA expression *in vitro*. IL-8 is commonly overexpressed in surgical specimens of pancreatic cancer tumor tissue (32,33), and expression levels correlate with metastatic potential and tumor growth (34,35). IL-8 promotes the growth of pancreatic tumors as a primary mediator of angiogenesis (32,36), and IL-8 expression levels have been observed to correlate with angiogenesis, tumorigenicity and metastasis in numerous xenograft and orthotopic *in vivo* tumor models, including pancreatic cancer models (34). COX-2 is almost undetectable in the majority of tissues under normal physiological conditions (37), although it is a markedly inducible molecule that is involved in proliferation and the inflammatory response (38,39). COX-2 and COX-2-derived prostaglandins have been observed to mediate tumor growth and metastasis in animal models by inducing the formation of blood vessels (40). The selective COX-2 inhibitor celecoxib has been previously reported to inhibit lung and lymph node metastasis in a colon cancer xenograft, and COX-2 inhibition by celecoxib has been observed to decrease angiogenesis, vascular endothelial growth factor expression levels and prostaglandin E_2_ production (41). Pioglitazone also suppresses COX-2 expression and inhibits the metastasis of colon cancer cells *in vivo* (26), and it may inhibit xenograft angiogenesis through the inhibition of IL-8 and COX-2 mRNA expression. In conclusion, pioglitazone may inhibit metastasis by the induction of cellular differentiation and a stable cell phenotype, and by inhibition of tumor cell dissociation from the primary tumor through antiangiogenic effects.

TZD has also been demonstrated to inhibit pancreatic cancer cell invasiveness, a process that affects gelatinolytic and fibrinolytic activity with a mechanism independent of PPAR-γ activation, which involves matrix metalloproteinase 2 and plasminogen activator inhibitor 1 expression (22). There may be several unknown mechanisms of pioglitazone-mediated metastasis inhibition. To clarify the precise mechanism of metastasis inhibition, further studies are required.

Type 2 diabetes is the most common form of diabetes and is associated with a higher risk of cancer (42). The association between diabetes and the risk of pancreatic cancer has been investigated for a long time. In addition, various preclinical and observational studies have revealed that antidiabetic medications may affect the risk of developing pancreatic cancer, although a meta-analysis of these studies did not reveal a protective or harmful association between antidiabetic therapies and the risk of pancreatic cancer in patients with diabetes (43). Recently, attention has focused on the associated between pioglitazone and an increased risk of bladder cancer. Laboratory animals have been shown to develop bladder tumors subsequent to the administration of experimental drugs with dual PPAR-α and PPAR-γ activity (44). Muraglitazar, a dual human PPAR-α/γ agonist, has been found to induce a dose-related increased incidence in transitional cell papilloma and carcinoma of the urinary bladder in male rats (45). These experimental data indicate that TZDs may be a risk factor for bladder cancer in patients with diabetes, but findings from clinical and epidemiological studies are inconsistent (46,47). A recent meta-analysis revealed that pioglitazone treatment appears to be associated with a significantly increased risk of bladder cancer in patients with diabetes (48). A current concern is whether the use of pioglitazone is associated with the risk of cancer. However, the association between TZD treatment and the risk of cancer remains controversial. The mechanisms underlying the pro-tumor potential of pioglitazone for bladder cancer are not yet fully understood.

As shown in the present study, pioglitazone may be useful to prevent pancreatic cancer metastases, particularly in cases of unresectable advanced disease in diabetic patients. However, careful attention is required with regard to the prophylactic use of pioglitazone following curative treatment due to the potential to induce another malignant tumor, such as bladder cancer. To confirm the usefulness of pioglitazone in pancreatic cancer treatment, clinical trials are warranted.

## Figures and Tables

**Table I:** Effect of pioglitazone on BxPC-3 xenografts (n=8).

	Median tumor weight (range), g		
		
Tissue	Vehicle	Pioglitazone	P-value
Rectal xenograft	1.67 (1.23–2.18)	1.38 (0.67–1.74)	0.046

Statistical analysis was performed using the Mann-Whitney U test.

**Table II:** Antimetastatic effect of pioglitazone in the BxPC-3 rectal xenografts.

	Median number of metastatic BxPC3 cells (range)^a^		
		
Target organ	Vehicle (n=8)	Pioglitazone (n=8)	P-value
Lymph node	7.07×10^4^ (0–3.29×10^5^)	0 (0–3.57×10^4^)	0.035
Lung	4.92×10^4^ (0–4.65×10^5^)	2.45×10^3^ (0–1.73×10^5^)	0.046

a Number of metastatic BxPC-3 cells in the organ was assessed by measurement of the human β-globin gene amplified by TaqMan polymerase chain reaction. Statistical analysis was conducted using the Mann-Whitney U test.
